# High-dimensional analysis reveals an immune atlas and novel neutrophil clusters in the lungs of model animals with *Actinobacillus pleuropneumoniae-*induced pneumonia

**DOI:** 10.1186/s13567-023-01207-4

**Published:** 2023-09-13

**Authors:** Na Li, Junhui Zhu, Peiru Chen, Chuntong Bao, Jun Wang, Tamim Abdelaal, Dexi Chen, Sibo Zhu, Wenjing Wang, Jiangnan Mao, Brendon P. Scicluna, Frits Koning, Fengyang Li, Liancheng Lei

**Affiliations:** 1 State Key Laboratory for Zoonotic Diseases, Key Laboratory of Zoonosis Research, Ministry of Education, College of Veterinary Medicine, Jilin University, Changchun, China; 2 Leiden Computational Biology Center, Leiden University Medical Center, Leiden, The Netherlands; 3 Department of Pattern Recognition and Bioinformatics Group, Delft University of Technology, Delft, The Netherlands; 4 Beijing Institute of Hepatology, Beijing Youan Hospital, Capital Medical University, Beijing, China; 5 School of Life Sciences, Fudan University, Shanghai, China; 6 Department of Applied Biomedical Science, Faculty of Health Sciences, Mater Dei Hospital, University of Malta, Msida, Malta; 7 Centre for Molecular Medicine and Biobanking, University of Malta, Msida, Malta; 8 Department of Immunology, Leiden University Medical Center, Leiden, The Netherlands; 9 College of Animal Science, Yangtze University, Jingzhou, Hubei China

**Keywords:** *Actinobacillus pleuropneumoniae*, immune response, bacterial pneumonia, neutrophils, subset, mass cytometry, piglet

## Abstract

Due to the increase in bacterial resistance, improving the anti-infectious immunity of the host is rapidly becoming a new strategy for the prevention and treatment of bacterial pneumonia. However, the specific lung immune responses and key immune cell subsets involved in bacterial infection are obscure. *Actinobacillus pleuropneumoniae* (APP) can cause porcine pleuropneumonia, a highly contagious respiratory disease that has caused severe economic losses in the swine industry. Here, using high-dimensional mass cytometry, the major immune cell repertoire in the lungs of mice with APP infection was profiled. Various phenotypically distinct neutrophil subsets and Ly-6C^+^ inflammatory monocytes/macrophages accumulated post-infection. Moreover, a linear differentiation trajectory from inactivated to activated to apoptotic neutrophils corresponded with the stages of uninfected, onset, and recovery of APP infection. CD14^+^ neutrophils, which mainly increased in number during the recovery stage of infection, were revealed to have a stronger ability to produce cytokines, especially IL-10 and IL-21, than their CD14^−^ counterparts. Importantly, MHC-II^+^ neutrophils with antigen-presenting cell features were identified, and their numbers increased in the lung after APP infection. Similar results were further confirmed in the lungs of piglets infected with APP and *Klebsiella pneumoniae* infection by using a single-cell RNA-seq technique. Additionally, a correlation analysis between cluster composition and the infection process yielded a dynamic and temporally associated immune landscape where key immune clusters, including previously unrecognized ones, marked various stages of infection. Thus, these results reveal the characteristics of key neutrophil clusters and provide a detailed understanding of the immune response to bacterial pneumonia.

**Supplementary Information:**

The online version contains supplementary material available at 10.1186/s13567-023-01207-4.

## Introduction

Pneumonia, an acute infection of the lungs that is commonly induced by bacteria, resulted in 2.5 million deaths in 2019 worldwide [1]. However, the prevention and treatment of bacterial pneumonia are difficult due to the continuous emergence of antimicrobial resistance, the lack of broad-spectrum vaccines, and variations in the pathogen virulence spectrum. The immune response induced by pathogens plays a key role in the development of pneumonia; however, little is known about the local immune response during bacterial infection. Therefore, to improve the prevention and treatment of bacterial pneumonia, it is essential to fully characterize the immune response in the lung during infection.

Although the role of several immune cell subsets in the development of bacterial pneumonia has been studied [2–6], a systemic analysis that simultaneously investigates innate and adaptive immune subsets in local lung tissues is currently lacking. Mass cytometry allows the simultaneous measurement of over 40 cellular markers at a single-cell resolution, providing the opportunity to investigate the immune response with unprecedented resolution in an unbiased and data-driven manner [7]. As the analysis methods for traditional flow cytometry are not suitable for high-dimensional mass cytometry datasets, novel computational algorithms have been developed, such as hierarchical stochastic neighbour embedding (HSNE) [8].

*Actinobacillus pleuropneumoniae* (APP) is a gram-negative bacterium that can cause porcine pleuropneumonia, a highly contagious and fatal respiratory disease that has caused high economic losses in the swine industry. APP substantially impacts pigs, which makes it an ideal animal model for drug interventional studies on pneumonia. Moreover, mouse models infected with APP have been shown to be suitable for the evaluation of potential therapeutic agents for pneumonia, as the lung displays typical pathological changes after APP infection, such as haemorrhagic, fibrinous, necrotizing pleurisy, and pneumonia [9–11].

Here, we applied high-dimensional mass cytometry to analyse the immune response in the murine lung during APP infection and found that distinct polymorphonuclear neutrophil (PMN) subsets and Ly-6C^+^ monocytes (Mo)/macrophages (Mφ) accumulated in the lungs of APP-infected mice. Moreover, a linear differentiation trajectory from inactivated to activated to apoptotic neutrophils emerged along with the infection process, namely, from the uninfected to onset to recovery phase. In addition, flow cytometry and single-cell RNA-seq analysis revealed that CD14^+^ PMNs were superior with regard to cytokine production ability, especially IL-10 and IL-21, compared to CD14^−^ PMNs in both mice and piglets. Strikingly, MHC-II^+^ or SLA-DQB1^+^ PMNs with antigen-presenting capacity were identified, and their numbers increased in the lung after APP and *Klebsiella pneumoniae* (KPN) infection in both mice and piglets. A correlation analysis between cluster composition and the infection process yielded a dynamic immune landscape and identified key immune clusters that marked the different stages of infection across tissues. Altogether, our data clarify the immune response after APP infection and lay the theoretical foundation for the development of new drugs and vaccines.

## Materials and methods

### Strain and culture of bacteria

APP serotype 5 reference strain L20 (APP 5b L20) was obtained from the Shanghai Entry-Exit Inspection and Quarantine Bureau (Shanghai, China) and grown in brain–heart infusion (BHI) broth supplemented with 15 μg/mL nicotinamide adenine dinucleotide (NAD) at 37 °C. For the mouse infection, an isolated colony was transferred to 3 mL of BHI medium and incubated for 6 h at 37 °C with 180 rpm agitation. The bacteria were centrifuged at 3500 × *g* and washed three times with phosphate-buffered saline (PBS).

### Experimental infection

Female ICR mice (18–22 g and 6–8 weeks old) were purchased from the Experimental Animal Center of Jilin University. ICR mice were randomly divided into two groups: the APP infection group (6.5 × 10^7^ CFU in 30 μL sterile saline per mouse, intranasally administered) and the control group (30 μL sterile saline per mouse, intranasally administered). Mice in the control group were anaesthetized by intraperitoneal injection of 150 μL of 1% pentobarbital sodium and sacrificed after weighing the body weight before infection. At 6, 12, 24, and/or 48 h after infection, as indicated in each experiment, the body weight was observed before sacrifice. The mice were sacrificed after anaesthesia, and lung and spleen tissues were collected for subsequent analysis. Lung index = the lung weight of the mouse/the body weight of the mouse. The lung weight increase rate = (the value of mouse lung weight in each infection group—the average value of mouse lung weight in the control group)/the average value of mouse lung weight in the control group. The calculation of the spleen index and spleen weight increase rate was similar to that of the lung.

Rongchang piglets (45 days) were purchased from the Experimental Animal Center of the Harbin Veterinary Research Institute, CAAS. Nine APP-free and KPN-free piglets were randomly divided into three groups: the control group (1 mL of sterile saline per piglet, intranasally administered, *N* = 3), the APP infection group (1 × 10^8^ CFU in 1 mL of sterile saline per piglet, intranasally administered, *N* = 3), and a KPN infection group (2 × 10^7^ CFU in 1 mL of sterile saline per piglet, intranasally administered, *N* = 3). All piglets were anaesthetized with an intravenous injection of sodium pentobarbital solution (25 mg/kg body weight) and sacrificed after a 3- (APP) or 4 day (KPN) infection. Lungs were immediately collected for subsequent cell isolation.

### Cell isolation

The murine lungs were cut into fine pieces and digested with 1 mL of tissue digestive fluid containing 0.3 mg/mL collagenase IV (Sigma‒Aldrich, St.145 Louis, MO, USA), 25 U/mL DNAse I (Solarbio Life Science, Beijing, China) and 5% foetal bovine serum (FBS) in RPMI (Biological Industries, Kibbutz Beit Haemek, Israel) for 30 min at 37 °C and filtered through a 70 μm nylon filter. The red blood cells were then further removed from the isolated cells using 2 mL/lung Red Blood Cell Lysis Buffer (Solarbio Life Science). All isolated cells were resuspended in staining buffer. The procedures for single-cell isolation from the piglet lung were similar to those from the mouse lung. A piece of the lung from the lesions was cut into small pieces and added to 5 mL of digestive solution. After following the steps in mouse cell isolation to obtain single cells, these cells were further centrifuged using OptiPrep separation medium (Axis-Shield, Dundee, UK) to remove dead cells. The isolated cells were immediately used in RNA-seq experiments.

### Mass cytometry antibody staining and data acquisition

The antibodies used for mass cytometry are listed in Additional file 1. Purified antibodies lacking carrier protein were conjugated with metal reporters using the MaxPar X8 antibody labelling kit (Fluidigm Science, South San Francisco, CA, USA). Procedures for mass cytometry antibody staining and data acquisition were carried out as previously reported [12]. Briefly, cells from the lung were first incubated with 0.5 mL of 2 μM Cell-ID Cisplatin to identify dead cells. Cells were then stained with the antibody cocktail for 30 min on ice. After staining, the cells were stained with 0.5 mL of 125 nM Cell-ID intercalator-Ir (Fluidigm Science) to label all the cells in Fix and Perm Buffer (Fluidigm Science) overnight at 4 °C. Finally, cells were acquired with the Helios mass cytometer, and data were normalized using EQ Four Element Calibration Beads (Fluidigm Science).

### Mass cytometry data analysis

Pooled data from individual live CD45^+^ cells that were individually gated using FlowJo software (version 10.5.3), as shown in Additional file 2, were sample-tagged and hyperbolic-arcsinh-transformed with a cofactor of 5 using Cytosplore^+HSNE^ software [8]. The hierarchical stochastic neighbour embedding (H-SNE) analysis was carried out with default settings (Perplexity: 30; iteration: 1000). All H-SNE and t-SNE plots and Gaussian mean-shift clustering-derived cell clusters were generated using Cytosplore. Clusters contain at least 100 cells. Hierarchical clustering of the phenotype heatmap was generated with Euclidean correlation and average linkage clustering in MATLAB 2015. The t-SNE maps were generated as previously reported [13]. Wanderlust analysis was carried out on cells that were selected with the CD14^−^Ly-6C^−^ PMN as starting points using Cyt in MATLAB. The Cluster t-SNE maps were performed as previously described [14]. Briefly, the data matrix with cluster frequencies of CD45^+^ cells in the individual samples was normalized and computed to select the top ten highest variance principal components as input for the t-SNE analysis. The clusters with similar profiles were grouped together. The bar graphs were created using GraphPad Prism 8 software.

### Antibodies and flow cytometry

The antibodies used for flow cytometry are listed in Additional file 3. For cytokine detection, single-cell suspensions from the lungs of APP-infected mice (24 h) were stimulated with 20 ng/mL PMA (Solarbio Life Science) and 1 μg/mL ionomycin (Shanghai Yuanye Bio-Technology Co., Ltd, Shanghai, China) for 6 h at 37 °C, and brefeldin A (Biolegend, San Diego, CA, USA) was added for the final 4 h. Cells were incubated with cytometry antibodies for 30–45 min at 4 ℃ for surface staining. After staining with Cyto-Fast™ Fix/Perm Buffer (Biolegend), cells were incubated with intracellular antibodies for 20 min at room temperature. For APP detection, the cells derived from the infected lung were first incubated with the cytometry antibodies for 30–45 min at 4 ℃ for surface staining. After fixation and permeabilization, cells were stained with pure APP antibody (made in-house) for 20 min at room temperature. After washing, a goat anti-rabbit IgG secondary antibody-PE (R&D Systems, Minneapolis, MN, USA) was further stained for 30 min at 4 °C. The data were acquired on a CytoFlex Cytometer (Beckman, Miami, FL, USA) or a FACSAria^™^ III cytometer (BD Bioscience, San Jose, CA, USA) and analysed using FlowJo V10 Software.

### Single-cell RNA sequencing

Single-cell RNA sequencing was performed on all cells derived from the piglet lung as previously described [12]. Briefly, cells were combined with oil, reagents and beads and loaded on a Chromium Single Cell Controller (10X Genomics, Pleasanton, CA, USA). Lysis and barcoded reverse transcription of polyadenylated mRNA from the single cells were conducted in each gel bead emulsion. The sequencing libraries were prepared using the Chromium instrument and the Single Cell 3’ Reagent kit (V1), and the transcripts were sequenced using a Hiseq3000 System (Illumina, San Diego, CA, USA).

### Single-cell RNA sequencing data analysis

The analysis of single-cell RNA sequencing data was performed using the single-cell RNA-seq package “Seurat” in R software [15]. The Seurat object was generated according to the criteria that at least 200 genes were expressed by each cell and that each gene was expressed by at least 3 cells. The data were then filtered by the following parameters: (i) unique gene count per cell > 300 and < 4000; and (ii) mitochondrial percentage of all genes < 10. After log-normalization, a PCA reduction analysis was conducted based on the 13,682 variable genes. Next, graph-based clustering detection and the t-SNE algorithm were applied to the top 15 PCA dimensions. The resolution for cluster identification was 0.2. Violin plots, t-SNE plots, and GO and KEGG analyses were conducted using the package “Seurat”.

### Statistics

The Kruskal‒Wallis test with Dunn’s test was used for multiple group comparisons after a normality test was conducted. The Mann‒Whitney test was used for two-group comparisons. *P* ≤ 0.05 and *P* ≤ 0.01 were considered to be significant, as indicated by an asterisk, and extremely significant, as indicated by two asterisks. Groups with a sample size of 2 were not included in the statistical analysis.

## Results

### Identification of major immune lineages in the lungs of APP-infected mice

**Figure 1:**
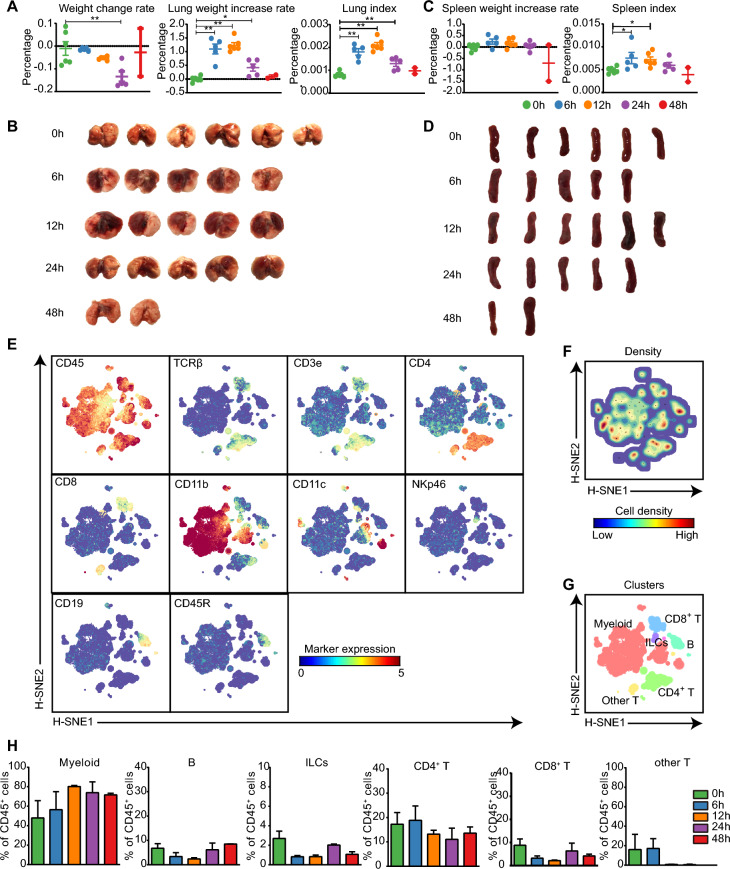
**Identification of major immune lineages in APP-infected lungs.** **A**, **B** Changes in lung weight change rate, lung weight increase rate, lung index (**A**), and pathological changes (**B**) at 6, 12, 24, and 48 h after APP infection compared to no infection (0 h). **C**, **D** Changes in spleen weight increase rate, spleen index (**C**), and pathological changes (**D**) at the indicated time points. For the lung analysis: 0 h: *N* = 6; 6 h: *N* = 5; 12 h: *N* = 5; 24 h: *N* = 5; 48 h: *N* = 2; for the spleen analysis: 0 h: *N* = 6; 6 h: *N* = 5; 12 h: *N* = 6; 24 h: *N* = 5; 48 h: N = 2. **E** HSNE embeddings of 219,967 immune cells derived from murine lung (*N* = 14) at the overview level. Each dot represents a landmark, and the size of the landmark is proportional to the number of cells it represents. Colours indicate the ArcSinh5-transformed expression value of each indicated marker. **F** HSNE plots show the cell density. **G** HSNE plots show the major immune lineage cluster partitions in different colours. **H** Cell frequencies of each major immune lineage in CD45^+^ cells during APP infection. All mass cytometric results were generated from 14 samples (0 h: *N* = 3; 6 h: *N* = 3; 12 h: *N* = 3; 24 h: *N* = 3; 48 h: *N* = 2) in one mass cytometry experiment.

To explore the lung immune response against APP infection, a mouse model including the onset to the recovery phase of APP infection was used as previously reported [9]. The weight change rate of mice dramatically decreased until 24 h after APP infection, and the lung weight increase rate and the lung index increased at 6 h and 12 h and decreased at 24 h and 48 h, suggesting that mice were severely infected at 6 h and 12 h and recovered from the infection after 12 h (Figure 1A). Similar results were obtained for the lung pathological lesions (Figure 1B). The spleen index, consistent with the lung index, was highest at 12 h, whereas no significant changes were observed in the spleen increase rate or pathological lesions (Figures 1C, D). To explore the immune landscape during the course of APP infection, we developed a mass cytometric panel consisting of 26-metal labelled antibodies (Additional file 1). This antibody panel allowed us to identify all of the major immune lineages, including myeloid cells, innate lymphoid cells (ILCs), CD4^+^ T cells, CD8^+^ T cells, other T cells and B cells, and investigate the heterogeneity within each lineage. After obtaining data from individual live CD45^+^ immune cells (Additional file 2), we pooled all of the data (219,967 CD45^+^ cells) derived from 14 lungs and carried out an HSNE analysis in Cytosplore [8]. Here, the landmarks described the lung immune composition (Figure 1E). Next, we determined the major immune lineages based on marker expression profiles and cell density features (Figures 1E, F), which were identified as CD3^−^CD19^−^NKp46^−^CD11b^+^/CD11c^+^ myeloid cells, CD3^−^CD19^−^CD11b^−^CD11c^−^ ILCs, CD3^+^TCRβ^+^CD4^+^ T cells, CD3^+^TCRβ^+^CD8^+^ T cells, CD3^+^TCRβ^+^CD4^+^CD8^+^ (other) T cells and CD19^+^ B cells (Figure 1G). Quantification of the percentage of each major lineage within each time point was then performed and revealed that the percentage of myeloid cells increased along with APP infection, while there were no significant changes in the other lineages (Figure 1H), indicating the key role of myeloid cells in the lungs upon APP infection.

### Distinct PMN clusters and Ly-6C^+^ inflammatory monocytes/macrophages accumulate in the lung post-APP infection

**Figure 2:**
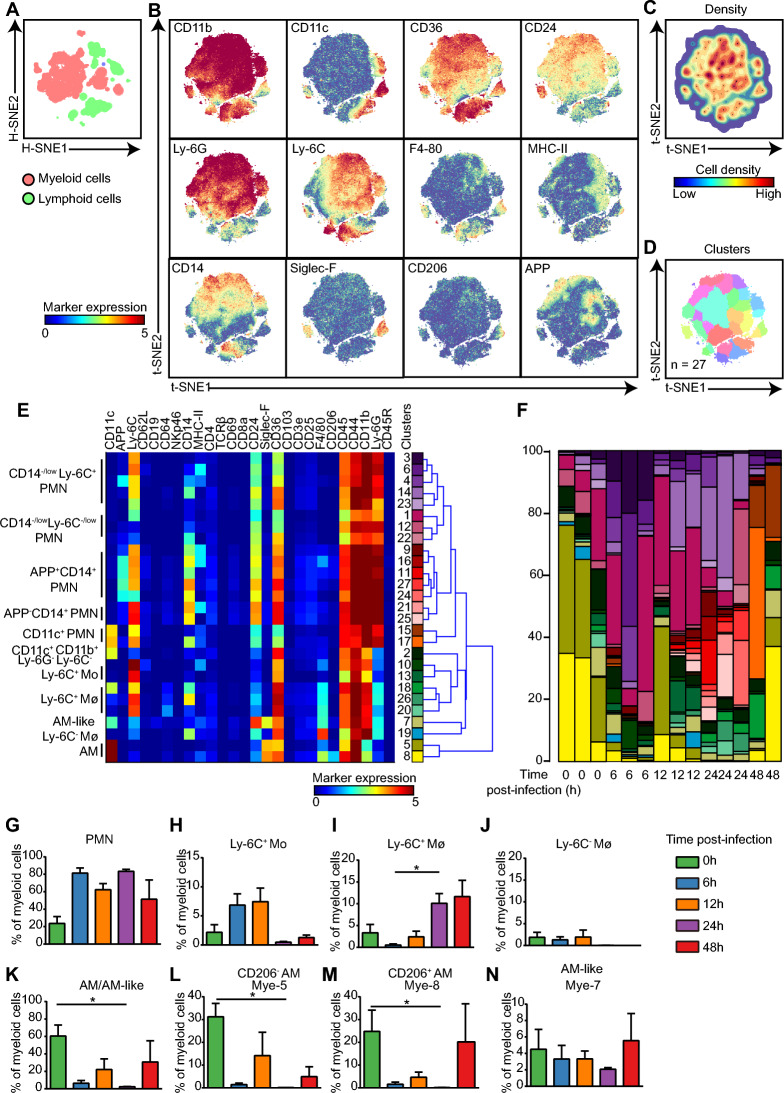
**Cluster identification in the myeloid cell compartment in the lung.** **A** An HSNE embedding of 219 967 immune cells derived from lungs (*N* = 14). Colours represent different immune lineages. **B** t-SNE embeddings of 148 820 myeloid cells show the ArcSinh5-transformed expression value of each indicated marker. **C** A density map shows the local probability density of the embedded cells. **D** A t-SNE plot shows cluster partitions. **E** A heatmap displays the median marker expression value and hierarchical clustering of the markers for 27 clusters identified in panel D. **F** Vertical bar graph depicting the composition of the myeloid cell compartment in each murine lung at the indicated time points. The coloured segment lengths represent the proportion of cells as a percentage of total myeloid cells in each sample. Colours as in panel E. **G**–**N** Cell frequencies of the indicated cell types in myeloid cells.

To further explore the pulmonary immune response, the myeloid cells were selected and embedded at the single-cell level (Figures 2A, B). Based on the density features of the embedded cells, we identified 27 phenotypically distinct myeloid cell clusters using Gaussian mean-shift clustering, each displaying a unique marker expression profile (Figures 2C, D). Unbiased hierarchical clustering of the heatmap further classified these cells into 11 major meta-clusters. Surprisingly, five major types of PMNs were identified based on the differential expression of CD14, Ly-6C, CD11c, and APP, while five types of Mos/Mφs, namely, alveolar macrophages (AMs), AM-like cells, Ly-6C^−^ Mφs, Ly-6C^+^ Mos and Ly-6C^+^ Mφs, were identified in the lung. Moreover, additional heterogeneity was observed due to the differential expression of MHC-II, the cell surface adhesion molecules CD44 and CD11b, and the B scavenger receptor CD36, especially in the PMN (Figure 2E). Strikingly, dendritic cells (DCs) were absent in the infected lung (Figure 2E). During the time course of infection, stacked bar analysis revealed dramatic changes in lung immune cell composition (Figure 2F). Consistent with a previous report [16], the percentages of both CD206^+^ and CD206^−^ AM dramatically decreased, while the percentages of all PMN subsets increased in the lung after APP infection (Figures 2G–N, Additional file 4). Moreover, during infection, the levels of Ly-6C^+^ Mos and Ly-6C^+^ Mφs in the lungs exhibited distinct temporal patterns, with the former increasing after 6 h and 12 h and the latter increasing at 24 h and 48 h (Figures 2H, I). Thus, these data reveal unprecedented heterogeneity in the myeloid cell populations and that distinct PMN clusters and Ly-6C^+^ Mo/Mφ accumulated in the lung at different stages of APP infection.

### A linear differentiation trajectory from inactivated to activated to apoptotic PMNs is formed during APP infection

**Figure 3:**
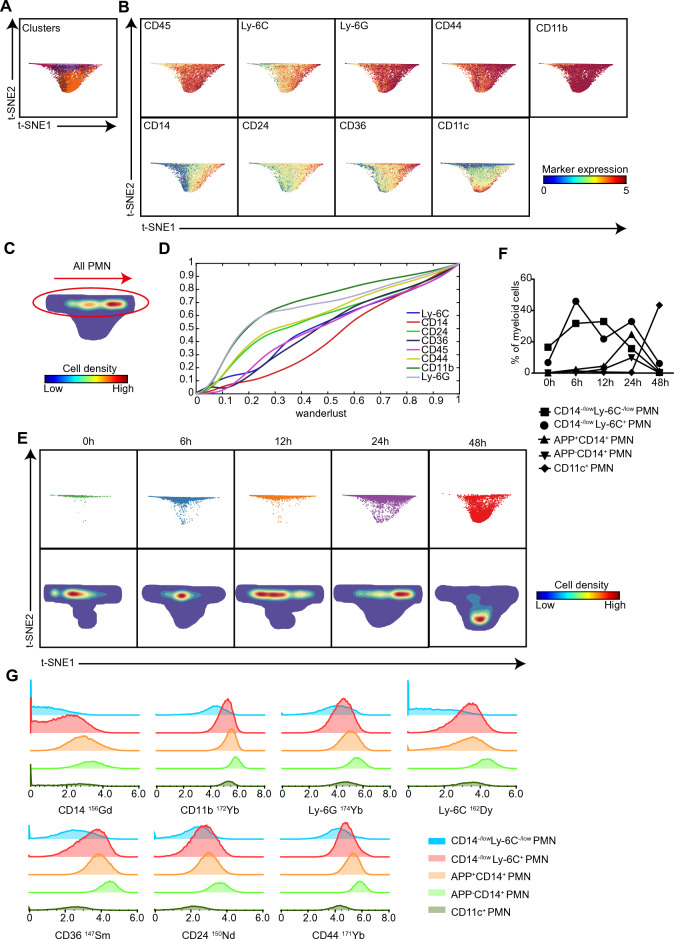
**Formation of a linear differentiation trajectory from inactivated to activated to apoptotic PMNs during APP infection.** **A** A t-SNE embedding of PMNs derived from lungs (*N* = 14). Colours represent distinct PMN clusters, as shown in Figure 2E. **B** t-SNE embeddings of PMNs show the ArcSinh5-transformed expression value of each indicated marker. **C** A density map shows the local probability density of the embedded PMN. Red encirclement indicates the cells in the linear trajectory. **D** Wanderlust graph showing the relative expression of the indicated markers along the differentiation trajectory, as indicated in panel C by the arrow. **E** t-SNE embeddings of PMNs derived from lungs (*N* = 14). Colours represent different time points of infection (upper row) and local density features of t-SNE-embedded cells (bottom row) at the indicated time points. **F** Cell frequencies of the indicated cell types in myeloid cells. **G** Overlay histograms show the indicated marker expression by five types of PMNs.

Based on the marker expression profiles, 17 distinct PMN clusters were identified in the lung post-APP infection (Figure 2E). To investigate the function of PMNs during infection, we performed a t-SNE analysis [13] to visualize the t-SNE computation dynamics of the PMNs. Interestingly, a linear trajectory among all PMNs except CD11c^+^ PMNs (Mye-15, Mye-17) was formed, where CD14^−/low^Ly-6C^−/low^ PMNs were next to CD14^−/low^Ly-6C^+^ PMNs, followed by CD14^+^ PMNs (Figures 3A, B). We next applied the Wanderlust algorithm [17] to determine the changes along the linear path, which revealed a gradual increase in the expression levels of the differentiation antigens CD45, Ly-6C and Ly-6G, adhesion-associated molecules CD44 and CD24, activation marker CD14, and apoptosis-associated markers CD24 and CD36 (Figures 3C, D). This finding indicated the formation of a linear trajectory from inactivated to activated to apoptotic PMNs during APP infection. Moreover, the PMNs were mainly in an inactivated state with a CD14^−/low^Ly-6C^−/low^ phenotype in the lung of uninfected mice, which started to express Ly-6C and became the dominant population after 6 h and 12 h of infection (Figures 3E–G). The levels of PMNs highly expressing CD14 started to increase post-12 h infection and reached the highest level at 24 h after infection. Importantly, these CD14^+^ PMNs expressed APP antigens and exhibited the characteristics of apoptotic cells, as they highly expressed CD24 and CD36, markers of apoptotic PMNs (Figures 3E–G), indicating that these cells play a key role in the recovery stages of infection. CD11c^+^ PMNs, which separated far away from other cells, were more abundant after 48 h of infection (Figures 3E–G). Thus, during the course of APP infection, a linear differentiation trajectory from inactivated to activated to apoptotic PMNs appeared along with stages of uninfected, onset, and recovery.

### CD14^+^ PMNs secrete both proinflammatory and anti-inflammatory cytokines

**Figure 4:**
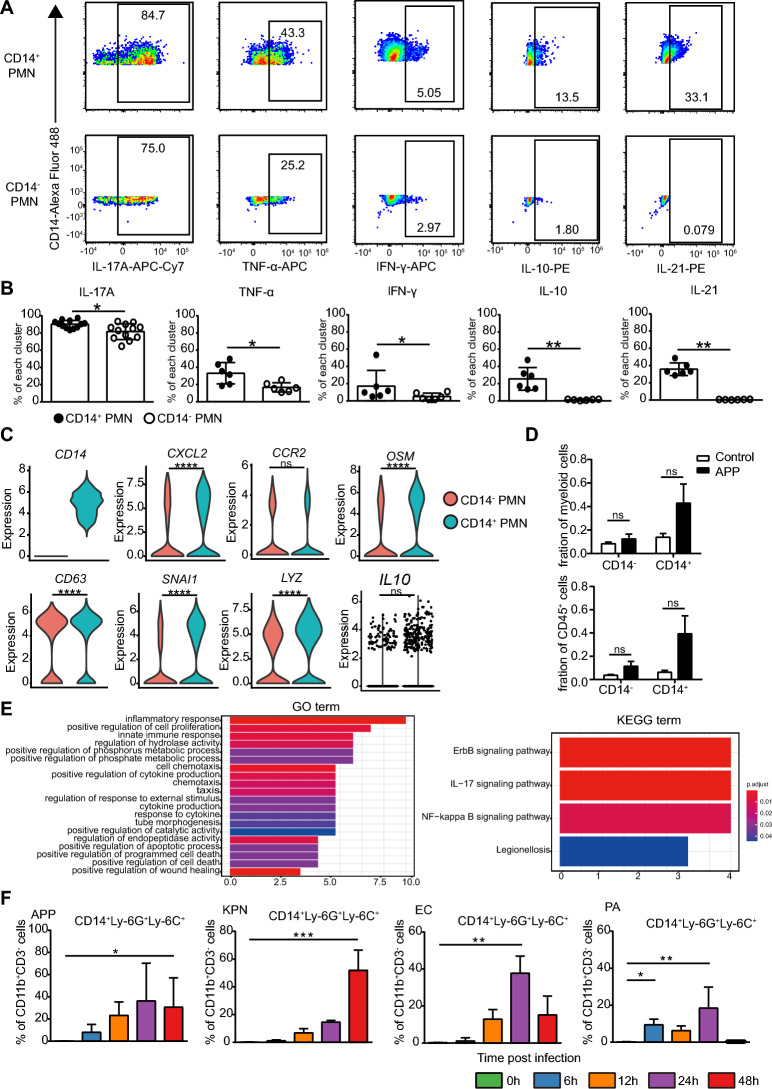
**CD14**^**+**^** PMNs in the lung produce more cytokines than their CD14**^**−**^** counterparts.** **A**, **B** Biaxial plots (**A**) from one representative experiment and bar charts (**B**) show the production of IL-17A, TNF-α, IFN-γ, IL-10, and IL-21 in CD14^−^ and CD14^+^ PMNs after stimulation with PMA and ionomycin. *N* = 6–12 samples in three independent experiments. Error bars indicate the mean ± SD. **C** Violin plots showing the RNA expression (log-normalized) of the indicated genes by CD14^+^ and CD14^−^ PMNs in the piglet lung after APP infection. **D** Bar plots show the frequencies of CD14^+^ and CD14^−^ PMNs in the piglet lung after APP infection. **E** GO and KEGG analyses show the main functions and signalling pathways of differentially expressed genes in CD14^+^ compared with CD14^−^ PMNs. All RNA-seq data were generated from one experiment with 3 samples per group. **F** The frequencies of CD14^+^ PMNs in the lungs after the indicated infection. *N* = 6 samples in three independent experiments.

The inflammatory response is strictly regulated by the coordination of pro- and anti-inflammatory mediator levels. Surprisingly, compared with their CD14^−^ counterparts, CD14^+^ PMNs expressed high levels of CD11b, CD44, Ly-6G, Ly-6C, CD24, and CD36, indicating the distinct functional properties of CD14^−^ and CD14^+^ PMNs (Figure 3G). To determine the functional profiles, we applied a traditional flow cytometry panel to assess the production of IL-17A, TNF-α, IFN-γ, IL-10, and IL-21 in CD14^−^ and CD14^+^ PMNs after stimulation with PMA and ionomycin. Both of these populations produced IL-17A, TNF-α, and IFN-γ. Significantly higher levels of these proinflammatory cytokines were observed in CD14^+^ PMNs than in CD14- PMNs. Importantly, dramatically higher frequencies of IL-10^+^ or IL-21^+^ cells were found in the CD14^+^ PMN compared to their CD14^−^ counterparts (Figures 4A, B), suggesting a greater anti-inflammatory function in the recovery stage of APP infection.

We next performed single-cell analysis on piglet lung cells after APP or KPN infection, where distinct PMN clusters were identified after unbiased clustering based on their gene expression profiles (Additional files 5A and B). PMNs derived from piglet lungs were then selected and subdivided into CD14^+^ and CD14^−^ PMNs in APP or KPN infection, respectively (Figure 4C, Additional file 5C). The frequencies of both subsets increased after APP or KPN infection (Figure 4D, Additional file 5D). Consistent with the flow cytometric data in mice, CD14^+^ PMNs expressed higher levels of *CXCL2*, *CCR2*, *SNAI1* (genes associated with PMN migration), *OSM* (a pleiotropic cytokine of the IL-6 group), *CD63* (a member of the tetraspanin superfamily of activation-linked cell surface antigens), and *LYZ* (a lysozyme) than CD14- PMNs (Figure 4C). Importantly, CD14^+^ PMNs also showed a higher mRNA level of *IL-10* than their CD14^−^ counterparts (Figure 4C). Gene Ontology (GO) and Kyoto Encyclopedia of Genes and Genomes (KEGG) enrichment analyses of differentially expressed genes in CD14^+^ PMNs revealed functions in the inflammatory response, innate immune response, cytokine production, cell chemotaxis, and ErbB, IL-17, and NF-κB signalling pathways (Figure 4E). Similar results were revealed in the piglet lung after KPN infection (Additional files 5C–E). Taking the stage at which these CD14^+^ PMNs appear into account, the results indicated that CD14^+^ PMNs had a stronger ability to produce cytokines than their negative counterparts and could also negatively regulate the immune response at the recovery stage of infection progression.

Finally, we explored whether the existence of CD14^+^ PMNs was universal in other bacterial-induced pneumonia models. Consistent with the APP or KPN infection, CD14^+^ PMNs were found in the lung after *E. coli* (EC) and *Propionibacterium acnes* (PA) infection (Figure 4F, Additional file 6). Moreover, these cells were more prevalent at 24 h or 48 h after infection (Figure 4F). Thus, CD14^+^ PMNs display a more active status.

### PMNs with antigen-presenting cell characteristics are identified and enriched in both porcine and murine lungs after APP or KPN infection

**Figure 5:**
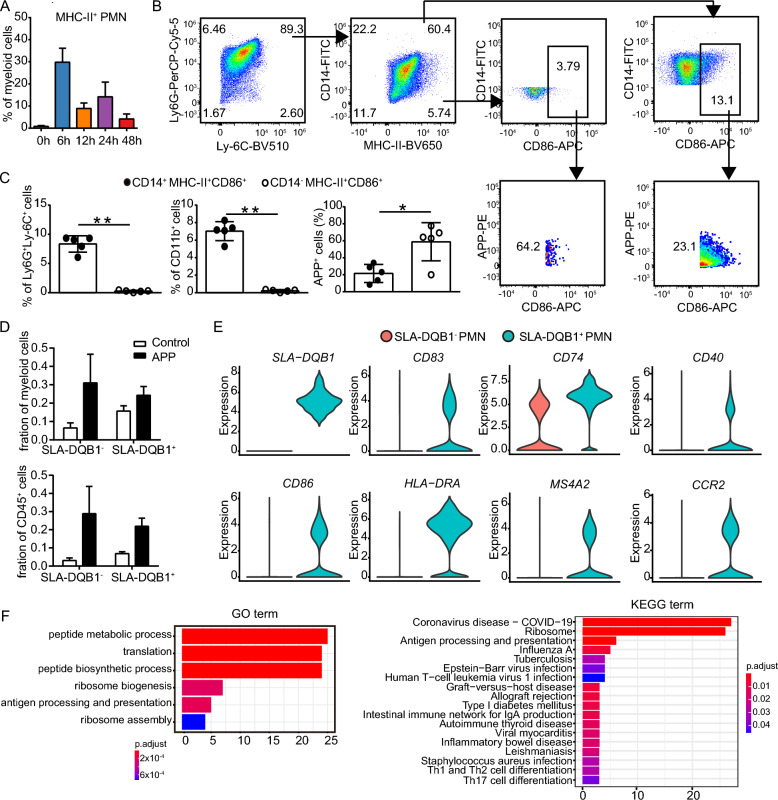
**Identification of APC-like PMNs in the lungs of mice and piglets after APP infection.** **A** Frequencies of MHC-II^+^ PMNs in the murine lung post APP infection. **B**, **C** Biaxial plots (**B**) from one representative experiment and bar charts (**C**) show the percentage of CD14^+^MHC-II^+^CD86^+^ or CD14^+^MHC-II^+^CD86^+^ cells within Ly-6G^+^ PMNs and the frequencies of APP^+^ cells within their parent cells. *N* = 5 samples in three independent experiments. Error bars indicate the mean ± SD. **D** Bar plots show the frequencies of SLA-DQB1^+^ and SLA-DQB1^−^ PMNs in the piglet lung after APP infection. **E** Violin plots show the RNA expression (log-normalized) of indicted genes by SLA-DQB1^+^ and SLA-DQB1^−^ PMNs in the piglet lung after APP infection. **F** GO and KEGG analyses show the main functions and signalling pathways enriched in the differentially expressed genes between SLA-DQB1^+^ PMNs and SLA-DQB1^−^ PMNs.

The mass cytometric data revealed that 5 PMN clusters expressed MHC-II. Several of these clusters even coexpressed APP antigens. The number of these MHC-II^+^ PMNs increased after APP infection, especially at 6 h in the lung (Figure 5A). Thus, we determined whether PMNs express molecules associated with the antigen-presenting process in the lung after bacterial infection. Flow cytometric analysis validated the existence of MHC-II^+^CD86^+^ PMNs in mouse lungs after APP infection. The percentage of CD14^+^MHC-II^+^CD86^+^ cells was significantly higher than that of CD14^−^MHC-II^+^CD86^+^ cells; however, the APP^+^ cell frequencies were higher in CD14^−^MHC-II^+^CD86^+^ cells than in their CD14^+^ counterparts (Figures 5B, C).

Consistent with the murine data, single-cell analysis of the piglet lung also found that the proportion of SLA-DQB1^+^ PMNs, which expressed the mature DC marker *CD83* and the costimulation molecules *CD86*, *HLA-DR*, *CD74* (MHC class II invariant chain, Ii), *CD40*, *MS4A2*, and *CCR2*, increased post APP infection (Figures 5D, E)*.* Moreover*,* GO and KEGG enrichment analysis revealed that the differentially expressed genes in SLA-DQB1^+^ PMNs were enriched in functions including peptide metabolic process, translation, peptide biosynthetic process, ribosome biogenesis, ribosome assembly, and antigen processing and presentation and KEGG signalling pathways such as those involved in coronavirus disease-COVID-19, ribosome, antigen processing and presenting, Th1 and Th2 cell differentiation, Th17 differentiation, influenza A, tuberculosis, and *Staphylococcus aureus* infection (Figure 5F). Similar results were obtained after analysing the lungs of KPN-infected piglets (Additional files 5F-H). Interestingly, the top KEGG pathway in SLA-DQB1^+^ PMNs was coronavirus disease-COVID-19, suggesting that this PMN subset might also play a curial role during coronavirus infection (Figure 5F, Additional file 5H). Thus, these data indicate the existence of PMN-like antigen-presenting cells after APP or KPN infection in both murine and porcine lungs.

### Integrated analysis of the entire immune system reveals the infection-associated networks of immune subsets in the lung

By applying a similar approach to myeloid cells in the lymphoid compartment, we identified 21 phenotypically distinct clusters (8 CD4^+^ T-cell clusters, 3 CD8^+^ T-cell clusters, 3 double-positive (DP) T-cell clusters, 4 B-cell clusters, 2 innate lymphoid cell (ILC) clusters, and 1 CD25^+^CD45^+^ cluster) in the lung (Additional files 7A–C). In addition to the well-known central memory T (T_cm_) and effector memory T (T_em_) cells, this analysis also identified three previously unrecognized T-cell clusters. One of them expressed the myeloid cell differentiation antigen Ly-6G, namely, Ly-6G^+^CD4^+^ T_em_ (lym-19), while the other two clusters were CD69^−^ DP T (lym-1) and CD69^+^ DP T (lym-10) (Additional file 7D). The latter expressed CD69, a marker for tissue-resident T cells. For the B cells and ILCs, there were relatively few clusters identified in the current analysis, likely due to the composition of the antibody panel where the majority of antibodies were designed to capture the heterogeneity in the myeloid counterparts.

**Figure 6:**
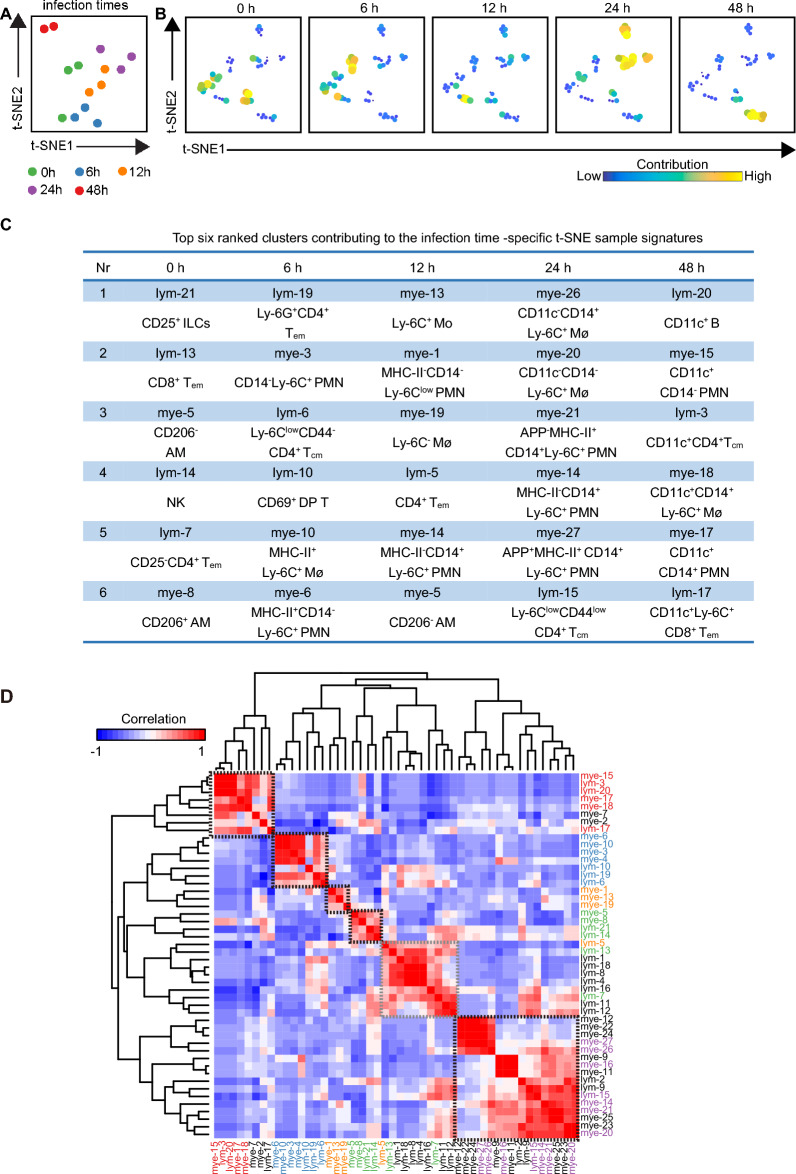
**Infection-associated immune clusters are identified in the lung.** **A** A t-SNE embedding of 14 lung samples, where the t-SNE was computed based on the cell frequencies of 48 immune clusters (% of CD45^+^ cells). One dot represents one sample. **B** t-SNE embeddings of 48 immune clusters from 14 samples. One dot represents one cluster. The size of the dot is proportional to the cell frequency value, which is more similar across tissues, and the clusters are closer. **C** A table depicts the top 6 clusters contributing to the infection-time-specific t-SNE signatures. **D** A heatmap shows the correlation among 48 immune clusters based on the cell frequencies of total CD45^+^ cells in each sample and hierarchical clustering. The top 6 clusters and the clusters that are significantly differentially enriched at different infection times are highlighted in different colours. Green: 0 h, yellow: 6 h, blue: 12 h, purple: 24 h, and red: 48 h.

To investigate the integrated immune profiles after APP infection, a t-SNE analysis was subsequently performed on all lung samples based on the cluster frequencies of CD45^+^ immune cells (Figures 6A–C). While the 0 h group was heterogeneous, this analysis revealed a clear progression trajectory where samples from 6 h were connected with those from 12 h, and samples from 12 h were then linked with the 24 h samples (Figure 6A). In contrast, the samples from 48 h clustered away from all other samples (Figure 6A). Thus, our results indicate the existence of an infection-associated progression pattern during the course of APP infection in the lung.

To reveal which cell clusters were strongly associated with the infection-associated trajectory, a second t-SNE analysis was performed on the same dataset to visualize the cellular networks by clustering the 48 clusters instead of the samples (Figure 6B). This analysis identified the top 6 clusters contributing to the infection time-specific t-SNE signatures (Figure 6C). In the control group, the major types contributing to lung signatures were innate immune cells, including CD206^−^ AM, CD206^+^ AM, CD25^+^ ILCs, and NK cells. After infection for 6 h, myeloid cells such as CD14^−^ PMNs (mye-3, mye-6) and activated inflammatory MHC-II^+^Ly-6C^+^ Mφs (mye-10) were dominant in the lung. Interestingly, cells contributing to the immune profile in the 6 h samples were T cells, including previously unrecognized Ly-6G^+^CD4^+^ T cells (lym-19, top) and CD69^+^ DP T cells (lym-10). Subsequently, various types of Mos, Mφs, and PMNs were enriched at 12 h and 24 h post-infection. Here, these cell types displayed a more activated status in the 24 h group than in the 12 h group, as the top clusters gradually expressed the activation marker CD14 or MHC-II. It is also worth noting that only CD14^+^Ly-6C^+^ PMNs, but not Mφs and other PMNs, were positive for APP antigens, suggesting a key role in the APP-induced immune response. For the 48 h samples, a diverse pool of CD11c-expressing B cells, PMNs, Mφs, and T cells contributed to the sample signatures (Figure 6C, Additional file 8). Next, the correlation analysis performed on all 48 clusters revealed strong infection time-specific patterns (black square) and further confirmed the top clusters identified in Figures 6C, D.

Altogether, our high-dimensional analysis of the immune response during the course of APP infection in the murine lung revealed a highly dynamic landscape of temporally associated and interconnected innate and adaptive immune cell cluster networks.

## Discussion

Developing effective strategies for the prevention and treatment of bacterial pneumonia remains difficult due to the increase in antibacterial resistance, lack of a broad-spectrum vaccine, and variations in the pathogen spectrum. Thus, it is urgent to develop effective vaccines and drugs for pneumonia by targeting the host immune response. The roles of several immune subsets derived from the peripheral blood of model animals infected bacterial pneumonia have been studied by traditional flow cytometry and immunohistochemistry, where the markers can be measured simultaneously, or by bulk RNA sequencing, which prevents single-cell level analysis [18–21]. Mass cytometry overcomes the above problems and can simultaneously analyse over 40 markers at a single-cell resolution, providing a unique opportunity to capture the dynamics of immune composition after infection in an unbiased data-driven manner [7, 22]. Here, we applied mass cytometry to dissect the immune cell response in the murine lung during APP infection. We built a dynamic immune blueprint of the lung and found that the clusters, such as PMN and Ly-6C^+^ Mo/Mφ, marked the infection stages. Moreover, during the infection, a linear differentiation path from inactivated to activated to apoptotic neutrophils was formed along with infection stages of uninfected, onset, and recovery. We further revealed that CD14^+^ PMNs produced more cytokines, especially IL-10 and IL-21, than CD14^−^ PMNs and that APC-like PMNs existed in both mice and piglets after both APP and KPN infection. Thus, our benchmark study provides, to our knowledge, initial clarification of the immune cell response induced by APP and highlights the key cellular clusters that engage in bacterial pneumonia.

In the lung, unprecedented heterogeneity in cellular patterns was revealed, including well-known and unknown key immune cells, such as ly 6G^+^CD4^+^ T cells and CD69^+^ DP cells. The specific functions of these cells warrant investigation in the future. Moreover, our results demonstrated the existence of infection-associated patterns along infection times, where the proportion of AMs was dramatically decreased after infection, and while PMNs and Mos quickly accumulated in the lung, as reported [23]. ILCs reside mainly in mucosal tissues and play a crucial role in lung inflammation, especially virus infection and allergy [24, 25]. A CD25^+^ILC2-like cluster (NKp46^−^CD3^−^CD25^+^ cells, lym-21) was enriched at 0 h and then decreased until 24 h, emphasizing the function of ILC2s in bacterial lung infection. Thus, these data highlight the importance of focusing on more sophisticated immune cell clusters.

PMNs are thought to be the main effector cells in bacterial infection through single-cell analysis and have recently been classified into 8 populations in the bone marrow, peripheral blood, and spleen during both homeostatic conditions and *E. coli* infection [26]. To our knowledge, our data were the first to reveal PMN heterogeneity in the lungs of healthy and APP-infected hosts, displaying unexpectedly pronounced heterogeneity that included 17 phenotypically distinct cell clusters. We not only found infiltrated PMNs in healthy individuals as previously described [27, 28] but also further confirmed that the subtypes of these cells were mainly MHC-II^−^CD14^−^Ly-6C^low^ PMNs and CD14^−^Ly-6C^low^ PMNs. During APP infection, the expression of Ly-6C, Ly-6G, CD44, CD14, CD24, and CD36 was gradually upregulated in these PMNs and eventually formed a linear differentiation path from inactivated to activated to apoptotic PMNs. Importantly, CD14^+^ PMNs expressed *BAX* and *APAF-1* in the porcine lung after APP or KPN infection (data not shown). Combined with the results of coexpressing APP antigen on CD14^+^CD36^+^CD24^+^ apoptotic PMNs, the data indicate that the phagocytosis of APP enhances neutrophil apoptosis via a mitochondrial pathway. Consistent with previous studies [29, 30], PMNs expressing CD14 were activated, producing high levels of cytokines and lysozyme in the lung. However, the level of anti-inflammatory IL-10 produced by CD14^+^ PMNs was dramatically higher than that produced by their CD14^−^ counterparts. As the CD14^+^ PMN subset was enriched at the recovery stage of infection, it may contribute to the resolution of infection by secreting anti-inflammatory cytokines and inducing apoptosis, which is then phagocytosed by macrophages.

A few reports have identified PMNs with antigen-presenting cell (APC) characteristics, mainly in the blood and bone marrow in mice and humans [31–33], supporting our identification of MHC-II^+^ PMNs in the blood but not in the spleen (data not shown). In contrast to the previously described PMN-DC-like cells [34, 35], MHC-II^+^ PMNs in our study expressed CD83 and CD86 but not CD11c, a marker expressed by DCs, indicating the distinction of our identified MHC-II^+^ PMNs. In addition, we found that both CD14^+^ and CD14^−^ PMNs had APC capacity and that the latter was stronger in the lung. Combined with the results showing that there were no DCs in the infected lung, these results suggest that CD14^−^ PMNs may act as APCs during APP infection in mice. It is worthwhile to note that for the first time, the functions of CD14^+^/CD14^−^ and APC-like PMNs were validated in the piglet lung after both APP and KPN infection at the transcriptional level through single-cell analysis, which suggests that the existence of these PMNs may be universal in bacterial pneumonia across species. IFN-γ and GM-CSF alone or in combination with other cytokines induced the acquisition of APC-like properties on PMNs derived from blood and bone marrow [32]; however, the factors that contributed to this process in the lung were obscure. Thus, the origin, differentiation, and function of PMNs with APC characteristics in homeostasis and disease need to be clarified.

It has been reported that adaptive immune cells such as T cells, especially memory T cells, may regulate innate immune cells at the early stage of infection [36–38]. Until now, the studies of T-cell function in APP infection were few and mainly focused on the chronic stage, as T cells are the traditional adaptive immune cells [20, 39]. In the spleen, the observation herein was that the majority of the top 6 clusters contributing to the infection-time-specific signatures were T and B cells (70% of all top clusters). In addition, several uncharacterized and newly identified cell clusters, such as Ly-6G^+^CD4^+^ T (lym-19, top one for 6 h samples), CD69^+^ DP T (lym-10), and CD11c^+^ B (lym-20, top one for 48 h samples) [40, 41], were identified in the lung. Functional analysis validated that Ly6C^+^Ly6G^+^CD4^+^ T cells cooperate with Mφ against APP-induced infection in mice (data not shown). CD69 is a marker for tissue-resident T cells, and it has been suggested that CD69^+^ DP T (lym-10) cells may be a local precursor for other T cells in the lung. These results further emphasize that more work is required to investigate adaptive immune cell function in the early stages of infection.

Although this research is one of the first to systemically study immune cell responses in bacterial pneumonia induced by APP using high-dimensional mass cytometry and single-cell RNA-seq analysis, the limitations need to be noted. First, our current panel was mainly designed for myeloid cells, leading to less heterogeneity in T cells, B cells, and ILCs. It would thus be important to include other lymphoid cell markers, cytokines, and chemokines to obtain more information on the lymphoid cell compartment and the functions of cell clusters. Second, 27 known and unknown cell clusters were identified in the lung; however, the functions of these clusters need to be clarified. Finally, the distinct functional properties of CD14^−^/CD14^+^ PMNs and APC-like PMNs were validated in APP or KPN infection. Nevertheless, it remains unclear whether these populations exist in infections by other bacteria, such as gram-positive bacteria.

In conclusion, we revealed a detailed and dynamic landscape of immune cell responses and identified the key infection-specific clusters in the lung during the course of APP infection. Notably, the study revealed that neutrophils are highly heterogeneous, with CD14^+^ PMNs producing high levels of both pro- and anti-inflammatory cytokines, and that APC-like PMNs exist in the murine and porcine lung post APP or KPN infection. Thus, our study clarifies the immune cell response in bacterial-induced pneumonia, which not only provides a dataset repository for the investigation of bacterial pneumonia but also lays the foundation for the future development of novel drugs and vaccines for respiratory infection by targeting the host immune system.

### Supplementary Information

**Additional file 1: Mass cytometry antibody list.** Table showing the antibodies used in the mass cytometry assays.

**Additional file 2:** **Mass cytometry analysis of the entire immune system in the murine lung.** **A)** Representative biaxial plots showing the gating strategy for individual live CD45^+^ immune cells with the percentage in the murine lung. **B)** Representative biaxial plots from one lung show the typical staining profiles of the mass cytometric antibodies used.

**Additional file 3: Flow cytometry antibody list.** Table showing the antibodies used for flow cytometry analysis.

**Additional file 4:** **Cell frequencies for identified clusters (% of myeloid cell compartments) in the lung during the course of APP infection.** Error bars indicate the mean ± SD.

**Additional file 5: Single-cell analysis of piglet lung cells after KPN infection.** **A)** A t-SNE embedding of the piglet lung cells shows the transcriptionally distinct clusters. The black circle indicates the PMN populations. Control (*N* = 3), APP infection (*N* = 3), KPN infection (*N* = 3). **B)** t-SNE embeddings of the piglet lung cells showing the indicated gene expression. Purple indicates high expression, and grey indicates no expression. **C)** Violin plots showing the RNA expression (log-normalized) of the indicated genes by CD14^+^ and CD14^-^ PMNs in the piglet lung after KPN infection. **D)** Bar plots show the frequencies of CD14^+^ and CD14^-^ PMNs in the piglet lung after KPN infection. **E)** GO and KEGG analyses show the main functions and signalling pathways of differentially expressed genes in CD14^+^ PMNs compared with CD14^-^ PMNs. **F)** Bar plots show the frequencies of SLA-DQB1^+^ and SLA-DQB1^-^ PMNs in the piglet lung after KPN infection. **G)** Violin plots show the RNA expression (log-normalized) of indicted genes by SLA-DQB1^+^ and SLA-DQB1^-^ PMNs in the piglet lung after KPN infection. **H)** GO and KEGG analyses show the main functions and signalling pathways of differentially expressed genes in SLA-DQB1^+^ PMNs compared with SLA-DQB1^-^PMNs.

**Additional file 6: CD14**^**+**^** PMNs are universal after gram-negative infection.** **A)** Representative biaxial plots showing the gating strategy for CD14^+^ PMNs using traditional flow cytometry. **B)** Representative biaxial plots showing the cell frequencies of CD14^+^ PMNs during the course of KPN infection.

**Additional file 7: Subset identification in the lymphoid cell compartment in the lung.** **A)** t-SNE embeddings of 71 147 lymphoid cells show the ArcSinh5-transformed expression value of each indicated marker. **B)** A density map shows the local probability density of the embedded cells. **C)** A t-SNE plot shows cluster partitions. **D)** Heatmap displaying the median marker expression value and hierarchical clustering of the markers for the 21 clusters identified in panel C.

**Additional file 8:** **Cell frequencies for identified clusters (% lymphoid cell compartments) in the lung during the course of APP infection.** Error bars indicate the mean ± SD.

## Data Availability

Mass cytometry data are available via Flow Repository (ID: FR-FCM-Z4SS). The other materials are available from the corresponding author upon reasonable request.
