# Supplementary material for: High-dimensional analysis reveals an immune atlas and novel neutrophil clusters in the lungs of model animals with Actinobacillus pleuropneumoniae-induced pneumonia
Source: Vet Res. 2023 Sep 13;54:76. doi: 10.1186/s13567-023-01207-4 (PMC10500746; doi:10.1186/s13567-023-01207-4)
Supplement: Supplementary file 1 — Additional file 1: Mass cytometry antibody list. Table showing the antibodies used in the mass cytometry assays. [file 13567_2023_1207_MOESM1_ESM.pdf]

Marker	Catalog No.	Source	Clone number	Metal isotope
CD45	3089005B	Fluidigm	30-F11	89Y
Siglec-F	155502	Biolegend	S17007L	141Pr
CD11c	3142003B	Fluidigm	N418	142Nd
TCRb	3143010B	Fluidigm	H57-597	143Nd
CD69	3145005B	Fluidigm	H1.2F3	145Nd
CD36	3147013B	Fluidigm	No.72-1	147Sm
CD4	100561	Biolegend	RM4-5	148Nd
CD103	121401	biolegend	2E7	149Sm
CD24	3150009B	Fluidigm	M1/69	150Nd
CD64	3151012B	Fluidigm	X54-5/7.1	151Eu
CD3e	3152004B	Fluidigm	145-2C11	152Sm
NKp46	3153006B	Fluidigm	29A1.4	153Sm
CD25 (IL-2R)	101913	Biolegend	3C7	154Sm
CD14	3156009B	Fluidigm	Sa14-2	156Gd
F4/80	3159009B	Fluidigm	BM8	159Tb
Ly-6C	3162014B	Fluidigm	HK1.4	162Dy
CD62L (L-selectin)	3164003B	Fluidigm	MEL-14	164Dy
CD19	3166015B	Fluidigm	6D5	166Er
CD8a	3168003B	Fluidigm	53-6.7	168Er
CD206 (MMR)	3169021B	Fluidigm	C068C2	169Tm
CD44	3171003B	Fluidigm	IM7	171Yb
CD11b (Mac-1)	3172012B	Fluidigm	M1/70	172Yb
Ly-6G	3141008B	Fluidigm	1A8	174Yb
MHC-II	ab55152	Abcam	6C6	175Yb
CD45R (B220)	3176002B	Fluidigm	RA3-6B2	176Yb
APP		Made in house	Poly-clonal	160Gd
