# Supplementary figures and images for: High-dimensional analysis reveals an immune atlas and novel neutrophil clusters in the lungs of model animals with Actinobacillus pleuropneumoniae-induced pneumonia
Source: Vet Res. 2023 Sep 13;54:76. doi: 10.1186/s13567-023-01207-4 (PMC10500746; doi:10.1186/s13567-023-01207-4)

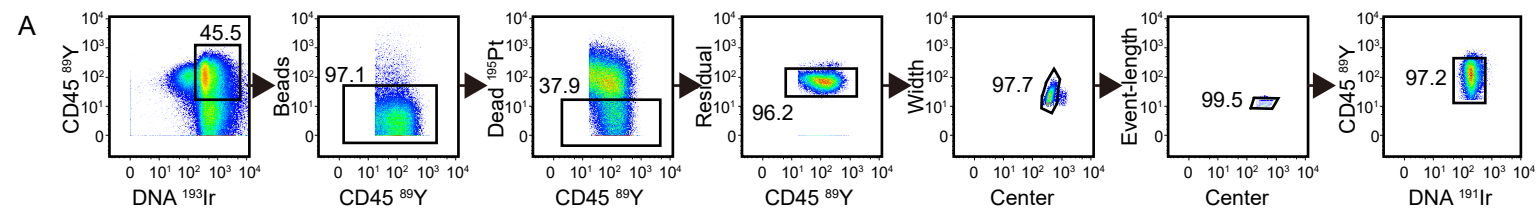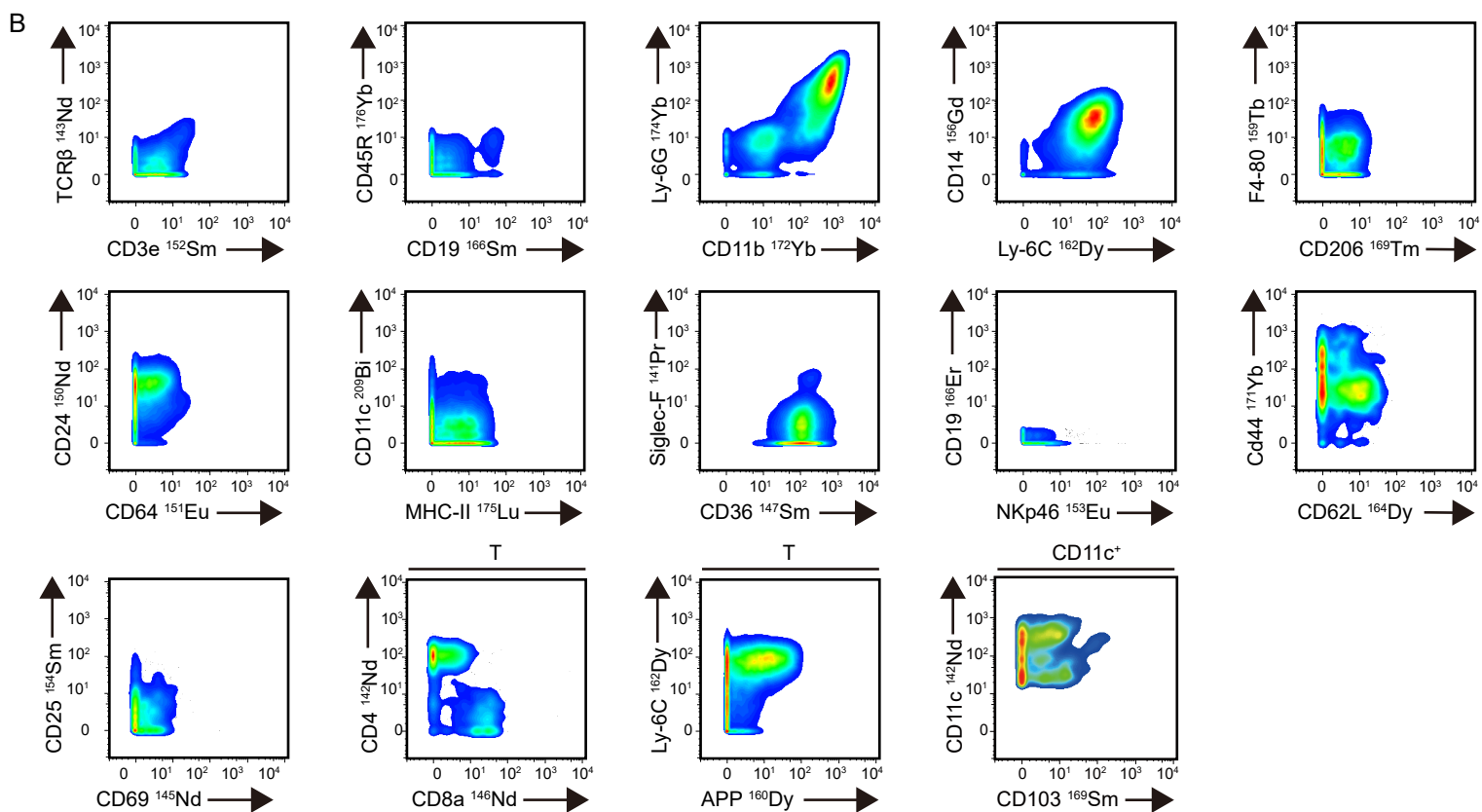

Supplement: Supplementary file 2 — Additional file 2: Mass cytometry analysis of the entire immune system in the murine lung. A) Representative biaxial plots showing the gating strategy for individual live CD45+ immune cells with the percentage in the murine lung. B) Representative biaxial plots from one lung show the typical staining profiles of the mass cytometric antibodies used. [file 13567_2023_1207_MOESM2_ESM.pdf]

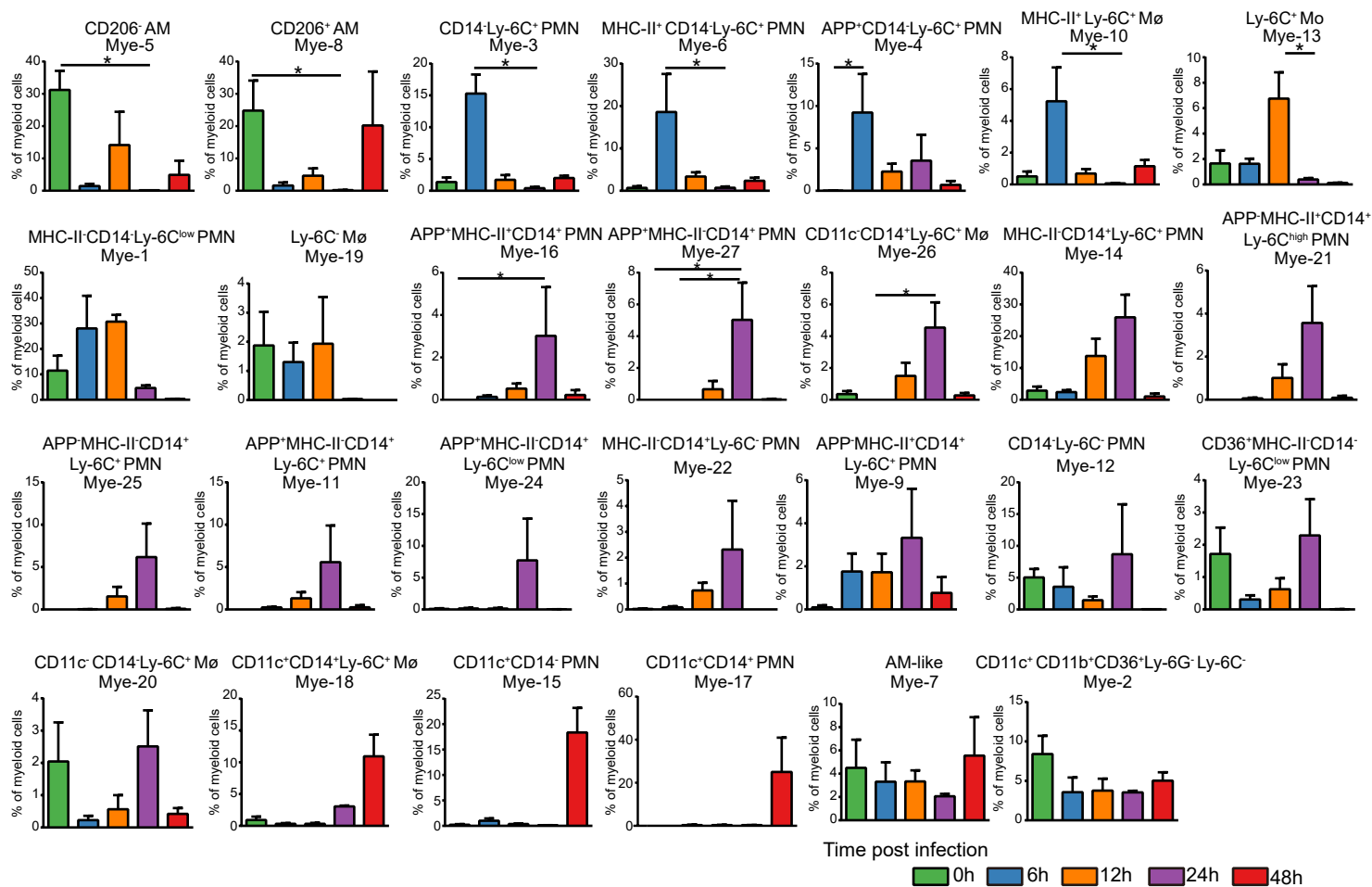

Supplement: Supplementary file 4 — Additional file 4: Cell frequencies for identified clusters (% of myeloid cell compartments) in the lung during the course of APP infection. Error bars indicate the mean ± SD. [file 13567_2023_1207_MOESM4_ESM.pdf]

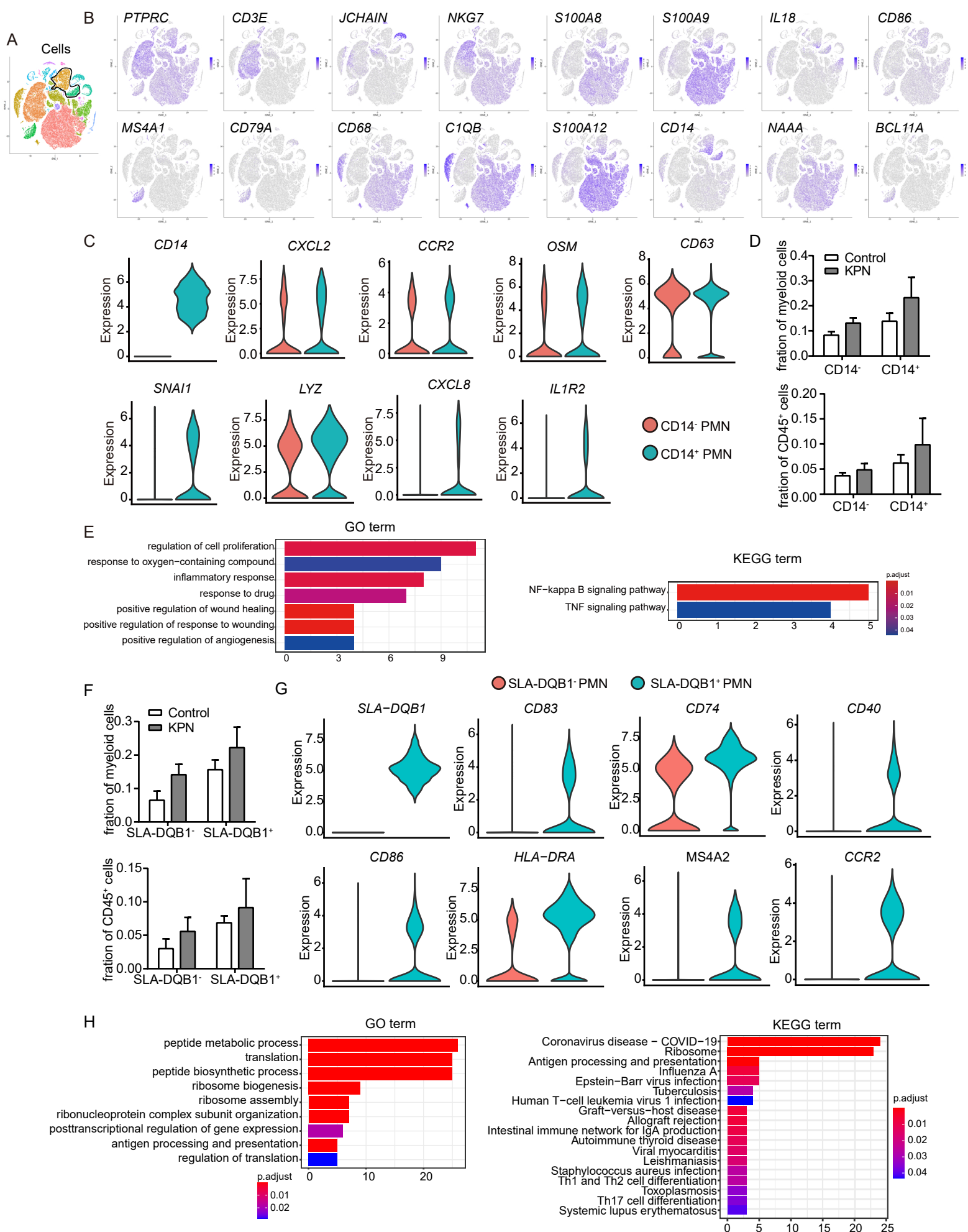

Supplement: Supplementary file 5 — Additional file 5: Single-cell analysis of piglet lung cells after KPN infection. A) A t-SNE embedding of the piglet lung cells shows the transcriptionally distinct clusters. The black circle indicates the PMN populations. Control (N = 3), APP infection (N = 3), KPN infection (N = 3). B) t-SNE embeddings of the piglet lung cells showing the indicated gene expression. Purple indicates high expression, and grey indicates no expression. C) Violin plots showing the RNA expression (log-normalized) of the indicated genes by CD14+ and CD14- PMNs in the piglet lung after KPN infection. D) Bar plots show the frequencies of CD14+ and CD14- PMNs in the piglet lung after KPN infection. E) GO and KEGG analyses show the main functions and signalling pathways of differentially expressed genes in CD14+ PMNs compared with CD14- PMNs. F) Bar plots show the frequencies of SLA-DQB1+ and SLA-DQB1- PMNs in the piglet lung after KPN infection. G) Violin plots show the RNA expression (log-normalized) of indicted genes by SLA-DQB1+ and SLA-DQB1- PMNs in the piglet lung after KPN infection. H) GO and KEGG analyses show the main functions and signalling pathways of differentially expressed genes in SLA-DQB1+ PMNs compared with SLA-DQB1-PMNs. [file 13567_2023_1207_MOESM5_ESM.pdf]

A

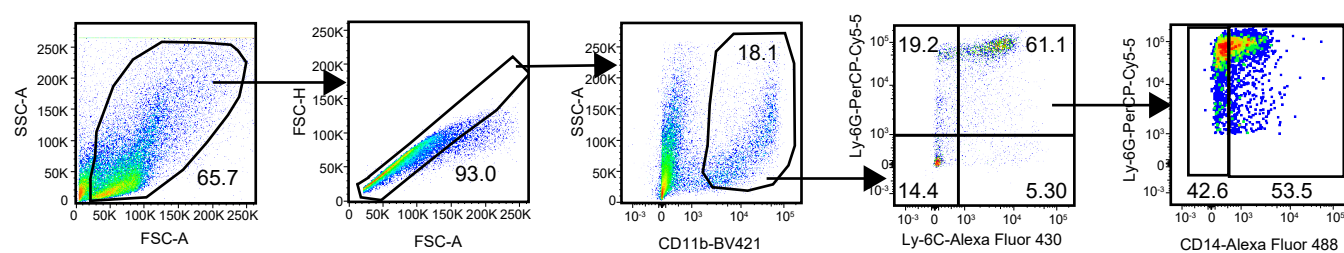

B

KPN infection

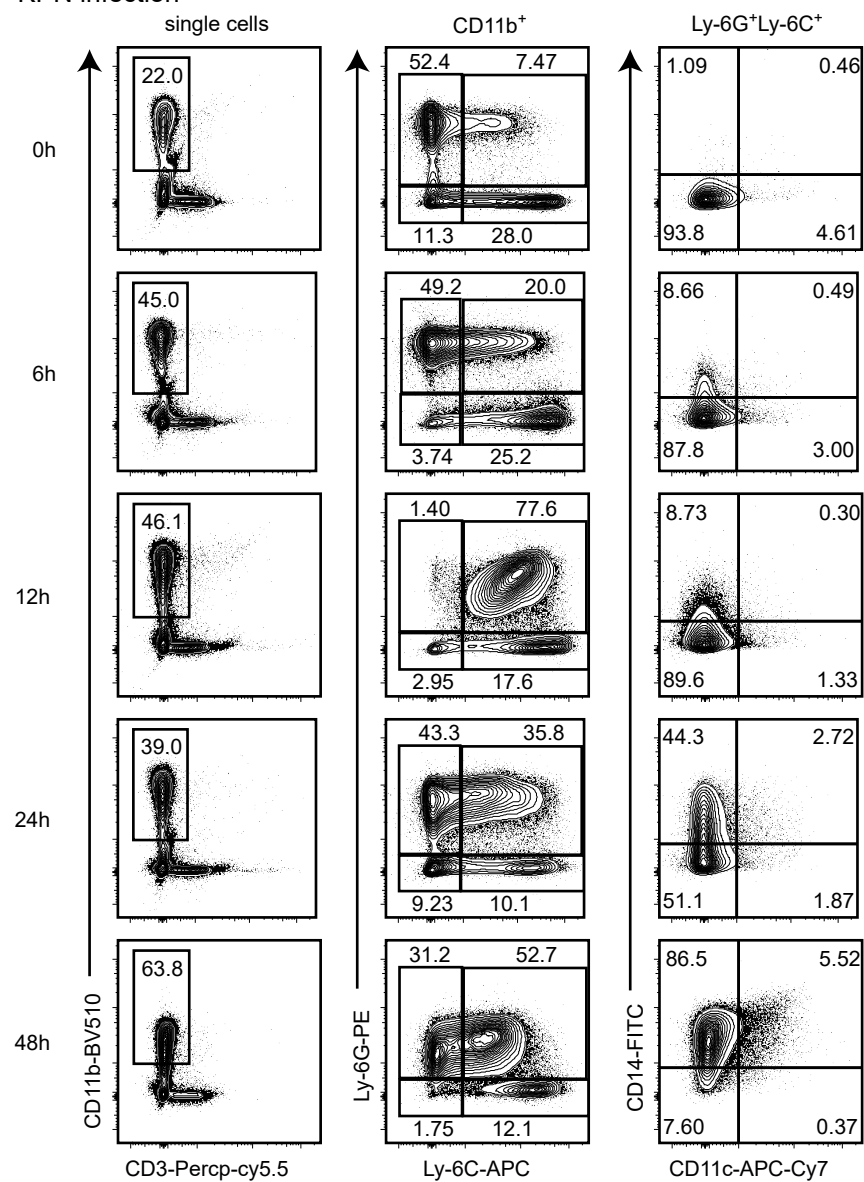

Supplement: Supplementary file 6 — Additional file 6: CD14+ PMNs are universal after gram-negative infection. A) Representative biaxial plots showing the gating strategy for CD14+ PMNs using traditional flow cytometry. B) Representative biaxial plots showing the cell frequencies of CD14+ PMNs during the course of KPN infection. [file 13567_2023_1207_MOESM6_ESM.pdf]

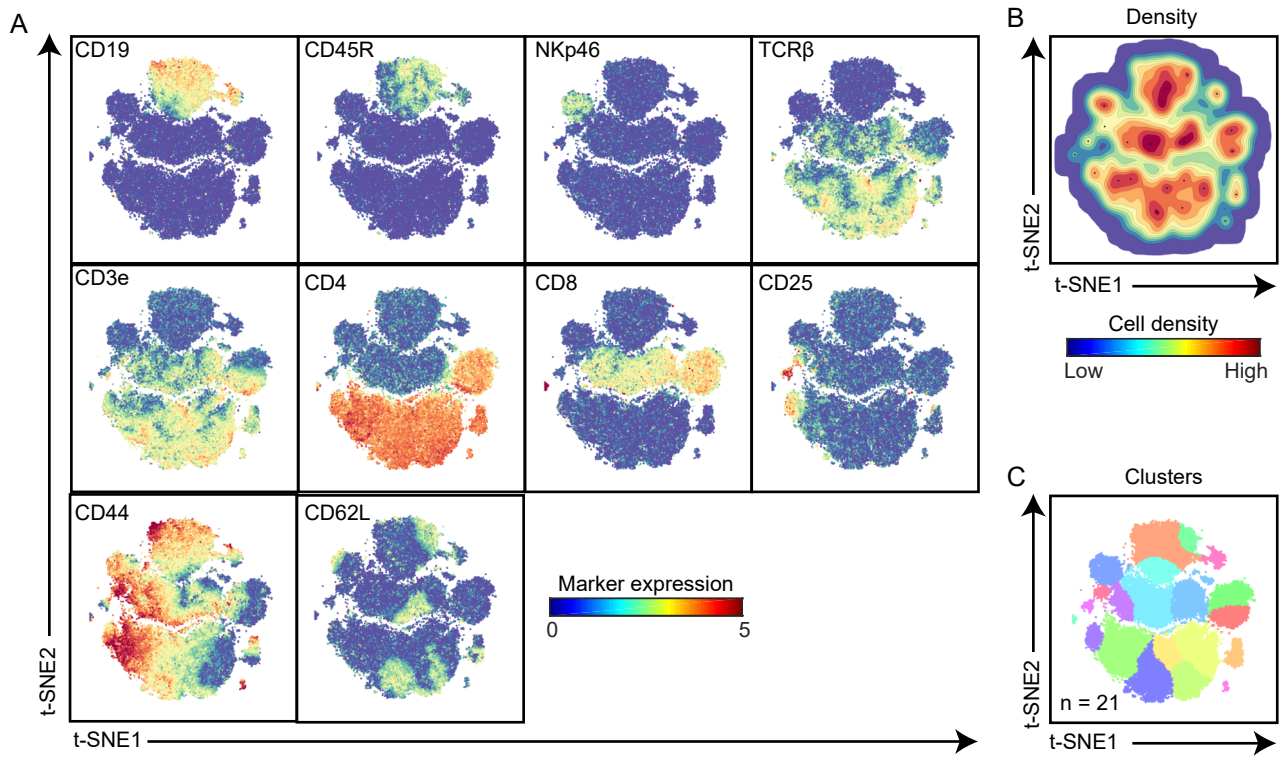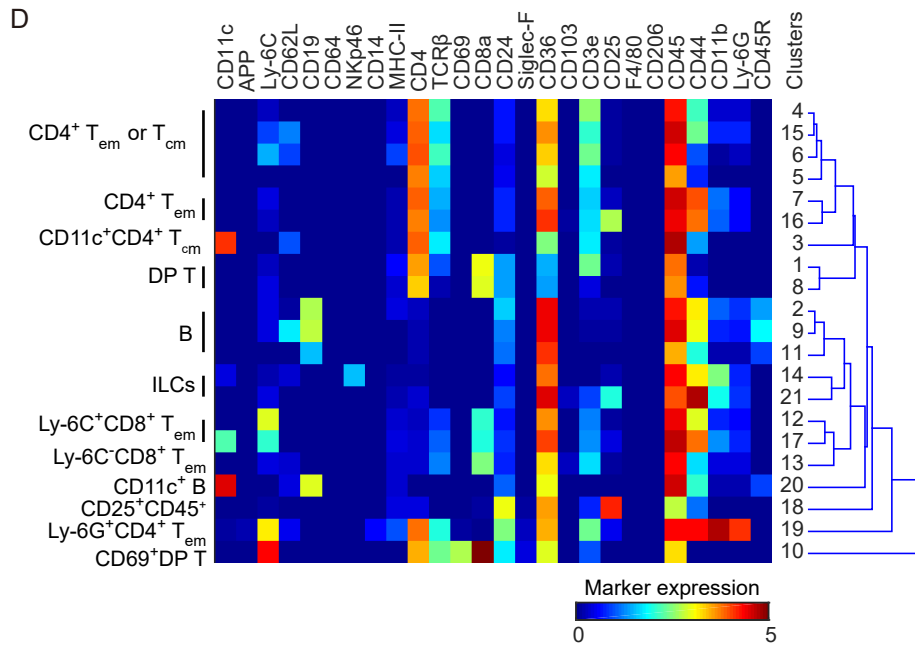

Supplement: Supplementary file 7 — Additional file 7: Subset identification in the lymphoid cell compartment in the lung. A) t-SNE embeddings of 71 147 lymphoid cells show the ArcSinh5-transformed expression value of each indicated marker. B) A density map shows the local probability density of the embedded cells. C) A t-SNE plot shows cluster partitions. D) Heatmap displaying the median marker expression value and hierarchical clustering of the markers for the 21 clusters identified in panel C. [file 13567_2023_1207_MOESM7_ESM.pdf]

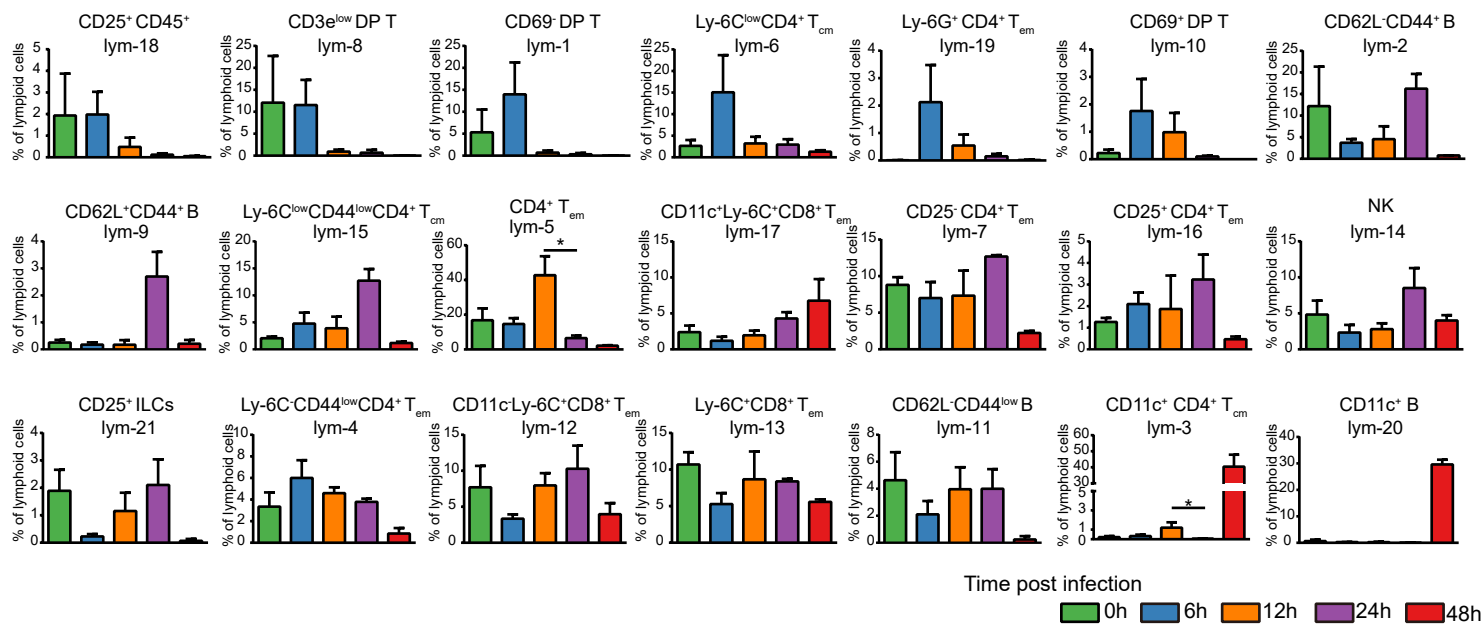

Supplement: Supplementary file 8 — Additional file 8: Cell frequencies for identified clusters (% lymphoid cell compartments) in the lung during the course of APP infection. Error bars indicate the mean ± SD. [file 13567_2023_1207_MOESM8_ESM.pdf]
