# Supplementary material for: High-dimensional analysis reveals an immune atlas and novel neutrophil clusters in the lungs of model animals with Actinobacillus pleuropneumoniae-induced pneumonia
Source: Vet Res. 2023 Sep 13;54:76. doi: 10.1186/s13567-023-01207-4 (PMC10500746; doi:10.1186/s13567-023-01207-4)
Supplement: Supplementary file 3 — Additional file 3: Flow cytometry antibody list. Table showing the antibodies used for flow cytometry analysis. [file 13567_2023_1207_MOESM3_ESM.pdf]

Marker	Catalog No.	Source	Clone number	Fluorochrome
CD11b	101236	Biolegend	M1/70	BrilliantViolet 421
CD14	123307	Biolegend	Sa14-2	FITC
Ly-6C	128033	Biolegend	HK1.4	Brilliant Violet 510
Ly-6G	46-9668-88	ebioscience	1A8-Ly6g	PerCP-eFluor 710
CD86	105012	Biolegend	GL-1	APC
MHC-II	107641	Biolegend	M5/114.15.2	Brilliant Violet 650
CD3	100217	Biolegend	17A2	Percp-Cy5.5
IL-17A	506940	Biolegend	TC11-18H10.1	APC/Cyanine7
TNF-α	506307	Biolegend	MP6-XT22	APC
IL-10	505007	Biolegend	JES5-16E3	PE
IFN-γ	505810	Biolegend	XMG1.2	APC
APP		Made in house	Poly-clonal	Pure
